# Prevalence of Acute Lower Respiratory Tract Infections due to Respiratory Syncytial Virus in Amirkola Children’s hospital, Northern Iran during March 2008-March 2010

**Published:** 2012-10-30

**Authors:** R Barari Sawadkohi, I Mohammadzade, A Mohammadpour-Mir, M Poor Nasrollah, M Valipour, F Hosseinzadeh, F Saeedi

**Affiliations:** 1 Pediatric infectious disease specialist, Infectious Diseases Research Center, Babol University of Medical Science, Babol, Iran; 2 Allergy and immunology specialist, Non- Communicable Pediatric Diseases Research Center, Amirkola Children’s Hospital, Babol University of Medical Science, Babol, Iran; 3 Pathologist, Non-Communicable pediatric diseases research center, Babol University of Medical Sciences, Babol, Iran; 4 General physician, Babol University of medical sciences, Babol, Iran; 5 Midwife, Non-Communicable Pediatric Diseases Research Center, Amirkola Children’s Hospital, Babol University of Medical Science, Babol, Iran; 6 Medical student, Student Research Committee,Babol University of Medical Science, Babol, Iran

Dear Editor,

Acute lower respiratory tract infections (ALRIs) are the main causes of morbidity and mortality in younger children accounting for 33-50% of mortalities in children less than 5 years of age, mostly in underdeveloped countries. Viruses are the most frequent causes of ALRIs and responsible for a considerable percentage of childhood deaths. The most important viruses are respiratory syncytial virus (RSV), influenza virus type A and B, parainfluenza virus 1-3 and adenovirus.(1) Human respiratory syncytial virus (HRSV), a genus Pneumovirus from Paramyxoviridae family, is the most common cause of respiratory infection in children specially younger than 6 months(3,4), and is considered to be the most important cause of viral bronchiolitis in young children.(5) RSV has seasonal distribution patterns as viruses circulate in winter and spring months with recurrent epidemics occurring in winter.(6) Also, RSV is known as a major causative agent of acute wheezing in children and RSV bronchiolitis in early childhood which leads to high rates of hospitalization. It is strongly associated with development of asthma, later in life.(7,8) Severe lower respiratory syndromes associated with RSV infection include pneumonia and bronchiolitis. Pneumonia is the leading cause of childhood mortality among children aged 5 years in all regions of the world, responsible for an estimated 19% of all deaths in this age group.(9) The aim of this study was to determine the prevalence of RSV-associated ALRI in children younger than 4 years hospitalized in Amirkola Children’s hospital affiliated to Babol University of medical sciences, northern Iran, since March 2008-2010.

This was a cross sectional descriptive-analytic study and 180 children aged <4 years admitted with clinical evidence of ALRIs were enrolled.

An episode of ALRI was defined as presence of cough, difficulty in breathing, and one or more of the following signs and symptoms: stridor, tachypnea, retraction, crackles and wheezing on lung auscultation. Informed consent was obtained from the parents. Plasma was collected from each patient (2mL) at the first 24 hours of admission and was transported to hospital’s laboratory and RSVs in samples were investigated by IgM antibody ELISA kit (IgM ELISA kit, IBL, Germany) with specificity and sensitivity >95%. Presence of virus antigen was approved indirectly by measuring the serum antibody titer and according to the manufacturer’s guideline, IgM>12 considered being positive.

Baseline data was obtained for the demographic characteristics, date, medical history, physical examination, blood culture, CBC, ESR, and hospital stay duration. Patients with bad or toxic general appearance at the time of admission, WBC>12.000 cell/mL, polymorphonuclears (PMN) rise, ESR>30mm/hour, CRP>10 mg/dL and isochronal positive cell culture were excluded from the study because these symptoms were in favor of bacterial pneumonia.

Data was analysis by χ2 test using SPSS version 17.0.

From 180 ALRI patients enrolled the study, 40 (22.2%) were RSV antibody positive and 140 (77.8%) were RSV antibody negative (serology was performed on samples from enrolled patients and 40 (22.2%) of the 180 ALRI infections were detected by serology as RSV).

RSV infection was most common among children aged 0–12 months (23.3%). It was also common for subsequent age of 13-24 months (20.5%), and 25-48 months (19.2%) (Table 1) but there was no significant relation between age and prevalence of RSVs (P=0.40).

**Table 1:** Frequencies of ALRIs and RSVs based on age, gender and season

Variables		RSV(%)	ALRIs(%)	P value
**Age (m)**	0-12	28	120 (66.7)	0.406
	13-24	(23.3)	34 (18.8)	
	25-48	7(20.5)	26 (14.5)	
		5(19.2)		
**Gender**	Male	19	111 (61.7)	0.112
	Female	(17.1)	69 (38.3)	
		21		
		(30.4)		
**Season**	Spring	14	62 (34.4)	0.22
	Summer	(22.6)	24 (3.13)	
	Autumn	4 (16.7)	33 (18.3)	
	Winter	6 (18.2)	61 (33.9)	
**Total**		16		
		(26.2)		
		40	180 (100)	-
		(22.2)		

RSV= Respiratory Syncytial Virus ALRI= Acute lower respiratory tract infections

The proportion of RSV+ females was higher than males but there was no significant relation between gender and prevalence of RSVs (P=0.11).

 The RSV epidemic peak occurred in winter and spring (respectively 26.2% and 22.6%). The lowest prevalence of RSV (16.7%) was seen in summer and there was a significant relation between season and prevalence of RSVs (P=0.22). There is a mounting interest in the hypothesis that RSV infection in the early childhood is an important risk factor for the subsequent development of recurrent wheezing and asthma later in life.(10) In this study, 180 children (61.7% males and 38.3% females) were studied for detection of RSV. RSV was detected in blood samples of 40 patients (22.2%). In Milani’s study on 365 children, RSV was detected in nasopharyngeal aspirate of 70 patients (19.18%) and it is similar to our findings.(11) Harish estimated that about 22% of all ALRIs occur due to RSVs.(12) Our finding showed prevalence of ALIRs associated RSV are similar to the world’s average.

Despite the presence of maternal antibodies, most hospitalizations occurred among infants aged <12 months, and nearly all children were infected by age 2 years. Like us, in Milani’s study, nearly all children infected with RSV aged less than 2 years.(11)

In our study, seasonal epidemic peak of RSV occurred first in winter and then in spring. Lan et al, found that RSV has a dramatic seasonal variation. Seasonal epidemic peak of RSV is mostly occurring in winter and early spring. So this study confirms our findings.(11,13) 

In conclusion, the most important limitations of this study were a relatively small sample size, inclusion of only hospitalized children and absence of highly sensitive diagnostic methods.

According to the high prevalence of childhood acute ALRI due to RSV, especially in winter and spring and in children less than 2 years of age, rapid and on time diagnosing of RSV using the immunofluorescence methods prevents using irregular antibiotic therapy and consequent drug resistances which is an increasingly spreading problem of patient’s treatment round the world.
